# Teachers’ Perceptions of Bullying in Saudi Arabian Primary Public Schools: A Small-Sample, Qualitative Case Study

**DOI:** 10.3390/children10121859

**Published:** 2023-11-27

**Authors:** Mohaned G. Abed, Lowai G. Abed, Todd K. Shackelford

**Affiliations:** 1 Department of Special Education, Faculty of Educational Graduate Studies, King Abdulaziz University, Jeddah 21589, Saudi Arabia; mabed@kau.edu.sa; 2 Department of Communication and Media Technology, College of Social Sciences and Media, University of Jeddah, Jeddah 21493, Saudi Arabia; lgabed@uj.edu.sa; 3 Department of Psychology, Oakland University, 654 Pioneer Drive, Rochester, MI 48306, USA

**Keywords:** bullying, communication, school, teacher, students, semi-structured interviews, parents, Saudi Arabia

## Abstract

Bullying among primary school students is a serious problem that often has multiple negative effects including poor academic performance and mental health problems. The current study used qualitative methodology to determine the role of communication in creating awareness and preventing bullying in a school setting through stakeholder intervention and bullying-prevention programs. If teachers are aware of bullying, then they are likely to take adequate measures to reduce or prevent future bullying. The researchers conducted semi-structured interviews with 15 teachers working in public primary schools in Jeddah, Saudi Arabia. The results provide an initial step in the Saudi Arabian context toward identifying the forms and types of school bullying, helping administrators, teachers, parents, and students reduce bullying and develop long-term plans for addressing bullying. Consideration of teachers’ perceptions may enable the development and implementation of new programs for addressing bullying in primary school students. The discussion highlights future research directions and the limitations of the current research.

## 1. Introduction

Bullying refers to the intentional and repeated harassment of an individual through physical, psychological, or verbal means [1,2,3]. The negative effects associated with bullying victimization include physical health problems [4] and mental health problems [5,6]. In addition, victims of bullying often become bullies themselves [7]. Bullying has attracted the attention of educators and policy makers in many parts of the world. Bullying often co-occurs with offensive words, and bullying typically has the aim of humiliating the victim while also attempting to impress peers. Bullying is usually intended to hurt or embarrass the victim [8,9]. Bullying can occur as physical, psychological, emotional, or internet-based aggression by students who use their power to harm weaker students [10,11,12]. Furthermore, research documents the negative impacts of bullying on the academic careers of victimized students [13,14]. 

Numerous studies indicate that bullying is prevalent in primary (i.e., elementary) schools [15]. Bullying will continue to negatively affect students and the quality of their education until measures are adopted to intervene and reduce or prevent it [16]. From a survey of more than 300,000 students in 48 developed and developing countries, Mullis et al. [17] found that more than 50% of students reported that they experienced bullying in school and 33% reported having been bullied weekly. Bullying has been documented in many countries, regardless of national wealth [18,19,20,21]. Bullying victimization at school may result in long-term social, emotional, and psychological effects [22,23], particularly for children with special educational needs [13,14,24]. 

The primary school setting is an important initial setting in education, as it includes the developmental phase during which personality traits emerge. It is a stage that impacts personality stability and balance and, therefore, bullying can result in adverse social and psychological effects on students. The negative effects of bullying may include struggling to develop social relationships, being unwilling to socialize with others, and having low self-esteem [25]. The negative effects also may extend to bullies themselves, who may as a consequence find it challenging to benefit from educational programs. School bullies also may progress to adolescent and adult criminality if measures are not adopted to address their bullying [13,14,25].

### 1.1. Literature Review

Bullying involves many people, including the bully, the victim, other learners, school counselors, parents, administrators, and teachers. Each individual can contribute to either reducing or facilitating bullying. Even though all of these individuals are involved, some may not realize their power to affect the situation and to address the problem behavior. In absolving themselves of responsibility, as is too often the case, each individual has a ready excuse. Victims may report that the bully is too powerful to stop. School authorities may report that they are unaware of bullying in their schools. The previous literature addresses links between bullying and aspects of schools, families, teacher characteristics, parental education, and students’ genders (references above, especially [1,2,3,4]). Less research has investigated the links between bullying and academic performance.

Communication (i.e., the transmission of information from one party to another, such as from teachers to students) can assist in preventing bullying or in alleviating the effects of bullying in primary schools. Some studies have addressed the role of stakeholders, including teachers, parents, and students, in preventing or reducing bullying. Snyder et al.’s [26] study indicates that school-based intervention programs can enhance communication and collaboration between parents and educators. However, only some schools have bullying intervention programs, and these must be implemented properly to maximize program effectiveness. 

Elmahdy et al.’s [27] study examined bullying in several countries, including Mexico and Saudi Arabia, among boys and girls at different education levels, including primary and tertiary. Data from Mexico indicated that the prevalence of bullying varied across education levels, occurring among 52% of elementary school students and 28% of tertiary school students. A representative sample of high school students in Saudi Arabia indicated a prevalence of 35% for bullying, in general, and 20% for physical violence, in particular. 

Communication has been cited as an effective tool to combat bullying, especially in primary schools in which younger students may be unable to take personal action compared to older students [28]. Snyder et al. [26] reported that the most effective anti-bullying programs engage three major stakeholders: parents, teachers, and students. In addition, support from parents and teachers can safeguard students’ well-being from the harmful effects of bullying [26]. Halah [29] established that students who communicate regularly with and feel empowered by their teachers are less likely to be perpetrators or victims of bullying. In addition, parents’ participation in school anti-bullying programs increased the success of those programs [29]. A meta-analysis found that parental participation in school activities is the most statistically significant factor in reducing school bullying [30]. 

Different anti-bullying programs highlight the role of communication in reducing or preventing bullying in schools [31]. However, no previous work has addressed anti-bullying programs implemented in Saudi Arabia. Studies in Western countries have addressed the use of online hashtags and their success in reducing or preventing bullying. These programs often use hashtags that highlight the role or importance of communication, including the #SpeakUp program and the #NoTrap program [26,31]. In the case of the #SpeakUp anti-bullying program of 2016, about 70% of students who reported that a teacher witnessed them being bullied and intervened indicated that the bullying stopped after the intervention [26]. This is consistent with the best practices, instructing victims and bystanders to report the matter or to involve a teacher or trusted adult when they experience bullying [26]. 

The meta-analysis by Gaffney et al. [30] showed that when students were asked which activities and resources helped them to address bullying, most indicated discussing the matter with a parent. The same study also noted that students reported feeling more confident speaking about bullying (with the goal of reducing the bullying) with their parents and teachers than speaking with their peers, siblings, counselors, staff, and other adults. This highlights the role of parents and teachers in alleviating bullying through communication [30]. De Luca et al. [32] found that parent/child discussion assisted with dealing with bullying, that teachers should intervene in bullying when it is reported to them, and that the parents of victims and perpetrators should be alerted when bullying occurs. The same study found that when teachers and parents become involved, the bullying situation improves significantly over time and rarely worsens. Snyder et al.’s [26] study on school bullying at different educational levels found that bullying situations improve when a teacher or administrator communicates with the perpetrator and victim, sends a message home to the parents, calls the parents, and initiates and participates in parent-teacher meetings about the bullying (see also chapters in [15]). 

The success in alleviating or preventing bullying in school depends on several factors, one of the most important of which is the teachers’ awareness of the bullying. Halah’s [29] study, conducted in Saudi Arabia, found that since primary school teachers in Saudi Arabia were often unaware of bullying, several strategies to prevent bullying were identified, including the need to develop and implement anti-bullying programs. Using a convergent parallel mixed-method design, the study found that fewer than half of the respondent teachers were aware of bullying and that those who were aware did not know how to treat students or address the situation [29]. Moreover, teachers did not employ anti-bullying programs and neither were they trained nor skilled to address bullying to maintain a safe school setting. 

Yet, anti-bullying programs have been found to be effective. For instance, Sainz and Martin-Moya [33] found that bullying-prevention programs helped to raise awareness of the problem within the school, improve the school environment, and decrease conflict and cases of bullying. Schools that participated in the study reported being highly satisfied with the results of the programs, suggesting that schools should consider a compulsory implementation of bullying-prevention programs. Gaffney et al.’s [30] meta-analysis discovered that creating awareness through anti-bullying programs is helpful for the entire school community because students can be involved in bullying not only as bullies and victims but also as bystanders and defenders. Thus, such programs help to reducing bullying. Zambuto et al. [31] found that most anti-bullying programs provided teachers with strategies for assisting students in developing strategies to resolve conflict positively. Moreover, most anti-bullying programs highlight communication (especially between students, parents, and teachers) as a solution to bullying or prevention of the same [31]. 

Teachers play a critical role in managing school bullying through adequate responses or intervention [32]. However, they must be equipped for this role by being alert to the situation and taking appropriate measures with the perpetrator and victim. De Luca et al. [32] also found that teachers’ competence in handling bullying influences students’ reports of bullying through a higher likelihood of teacher intervention after the bullying occurs.

### 1.2. Aims of the Research

The current study aimed to determine the significance of communication in creating awareness and preventing bullying in public schools in Saudi Arabia. In the following sections, we present a brief review of several relevant studies, we present the conduct of the current qualitative study, and we close with a discussion of the key findings of the current study.

### 1.3. Research Questions

The following literature review is guided by the following questions:

1. What is the significance of communication in creating awareness and preventing bullying in schools?
2. To what extent are Saudi Arabian primary school teachers aware of bullying and what interventions have they applied, if any?
3. Can school stakeholders’ (teachers, students, and parents) collaboration through communication reduce or prevent bullying in schools?
4. Do Saudi Arabian schools apply anti-bullying programs? If not, why not?

## 2. Methods

### 2.1. Design

The current study used a qualitative design methodology. The specific method used for data collection was the semi-structured interview, in which participants shared their perspectives on the relevant issues in narrative detail. Each interview lasted about 35 min. All interviews were conducted during early 2023 and in Arabic by the second author, a senior educational researcher with decades of experience in conducting qualitative, semi-structured and structured interviews with teachers, parents, students, and administrators in various educational settings in Saudia Arabia. After gaining the consent of the participants, the interviews were recorded and then transcribed verbatim. Respondents were asked to provide comments on the transcript, including clarifying or adding points where needed. This qualitative methodology was selected because of its ability to gather rich, personal, and detailed responses. Questions were crafted to allow teachers to express their perceptions of bullying in public primary schools in Saudi Arabia. The specific interview questions were: (1) Who is a bully, and what is bullying? (2) What are the causes of bullying, and what are its types? (3) Does bullying differ between the sexes? (4) What are the effects of bullying on the child and their academic achievement? (5) What is the role of the school in detecting bullying, confronting it, and reducing it? Question five was of significance because it was linked to the role of communication in creating awareness of bullying in schools and inviting teachers’ and parents’ interventions. 

### 2.2. Participants

The sample comprised 15 men teaching in mainstream government primary schools in Saudi Arabia in different municipalities in Jeddah. The participants taught children between 6 and 12 years of age, following the national curriculum in environments that are similar with regard to applicable laws and regulations. Convenience sampling was employed to recruit the participants. The available demographic data for participants was as follows: for teaching experience, 8 participants reported 1–10 years of experience, 4 participants reported 11–20 years of experience, and 3 participants reported 21–30 years of experience. For educational background, 13 participants had completed a Bachelor’s degree and 2 participants had completed a Master’s degree. Finally, participants reported the following ages (in years; frequency in parenthesis): 25–30 (3), 31–35 (5), 36–40 (3), 41–45 (2), 46–50 (1), and 51–55 (1).

The researcher initially approached potential respondents through their head teachers. The respondents were not offered any rewards, and none of the respondents withdrew from the study. 

### 2.3. Procedures and Materials 

Before the study began, the researcher secured ethical approval, permission, and collaboration from King Abdulaziz University. Once the participants had provided verbal consent to participate, the researcher asked them to sign a statement of informed consent. The researcher guaranteed the privacy and confidentiality of all responses at every phase of the data collection process. The researcher audio-recorded each interview using a digital device. Before they were translated into English, the interviews were transcribed verbatim by a locally sourced translator. Thematic analysis was conducted using mixed descriptive and inductive methods based on a computer software program available with NVivo software (version 1.0). 

## 3. Results

### 3.1. Defining a Bully and Bullying Behaviors 

Participants agreed that bullying is a type of harm committed against a child on a continuous and deliberate basis. They also agreed with the research definition that bullying behaviors include verbal aggression, spreading rumours, social rejection, and isolation. For example, according to Teacher 5 (T5), aggressive behavior, the desire to control the lives and behaviors of others, and the use of force with the aim of directly or indirectly harming others are prominent bullying behaviors. As T8 commented: “The bully is usually a selfish person. He deals with dictatorship and the logic of one opinion. He uses tyranny, threats and intimidation. He does not care about the feelings and opinions of others, and lacks empathy”. From T9’s point of view, there is no child who has not been bullied or harassed by a sibling or friend. Between pranks and petty teasing, bullies use their power (whether physical, or by use of knowledge of sensitive or embarrassing information) to control or hurt others. According to T1, a bully is a child who finds satisfaction in making fun of other children and hurting them verbally or through violence to prove to peers that the bully is stronger or more powerful than the victim. 

### 3.2. Types and Causes of Bullying 

Bullying among children is unwanted behavior and the teachers reported that it may be the result of something wrong with the bullying child. For example, a child who becomes a bully may have been neglected by his or her parents. They may also be a victim of domestic problems, domestic violence, or poor education. Several teachers noted that it is common for bullies to be victims of bullying themselves, which may make them feel angry and worthless, and thus they use bullying as a means of exacting revenge. If a child becomes accustomed to bullying between the ages of one and six years (the pre-school stage), he may suffer from the effects of this bullying throughout his time in primary, middle, and high school if there is no adult intervention.

From the teacher responses in this study, even those children who do not have problems originating at home can acquire bullying behaviors at school. This is a view acknowledged by T15, who said: “You can bully me with the popularity I lack. So this power difference makes it difficult for me to stand up for myself”. T3, T4, and T7 reported that this is true especially for children with disabilities. These children are at increased risk of psychosocial problems as a result of bullying (T3, T4, and T7). Many students with disabilities have been bullied at school, and being bullied is associated with difficulties in making friends (T7). From the point of view of T6, bullying in children can be attributed to a difficult upbringing, including neglect, cruelty, or excessive pampering.

T12 commented that parents or guardians are the ones who raise the bullying child, and it is possible that the bully acquired his behavior unintentionally from parents or guardians through the way they treated him, such as with threats and punishment. T12 further commented that difficulties or uncertainties in family life, such as experiences with divorce and domestic violence, may make it difficult for the child to deal successfully with life, and this plays a role in the child’s psychological state and may lead to feelings of insecurity. According to T12, such feelings can lead to hostility and bullying. 

However, some participants offered a different point of view than was expressed by the comments of T6 and T12. T13, for example, suggested that although domestic violence or exposure to the aggressive behavior of parents may be a cause of school bullying by a child, this is not an inevitable outcome of a difficult family life. If the child is educated in a school that adopts an anti-bullying program, and if there is a climate that supports them achieving this goal, T13 commented that bullying behavior might be prevented or reduced. T14 concurred with this sentiment, noting that, “The school environment is the main reason”. T14 further commented that the absence of deterrence and order in school can facilitate the spread of school bullying. Across the 15 teachers, there was broad agreement that bullying can be divided into the following categories: (1) verbal bullying includes threats, insults, and sarcasm; (2) physical bullying includes destroying things, kicking, punching, and hitting; (3) social bullying, includes spreading rumors about the child or deliberately excluding or neglecting a child; (4) relational bullying, sometimes referred to as psychological or emotional bullying, includes stalking, staring, and manipulating the child into believing that the alleged bullying is only happening in his or her mind; (5) and cyberbullying includes online taunts and threats, texting with comments on social media, or hacking online accounts.

### 3.3. Bullying by Boys and Girls

All of the teachers in the present study noted that bullying can take different forms in boys and girls. For instance, T15 said, “I think bullying in verbal form is most frequently present in the relationships between girls, while boys are involved more in physical violence”. T10 stated that “Using physical aggression can be considered a more evident profile for boys”. 

### 3.4. The Impact of Bullying on Children

All participating teachers agreed that victims of bullying can suffer from long-term behavioral or emotional problems. Specific problems observed by the teachers included low self-esteem, anxiety, depression, loneliness, and introversion, and several teachers also noted that victims of bullying are prone to aggressive behaviors and actions related to seeking revenge against bullies. Many teachers noted that victims of bullying often withdraw from social activities until they become silent and withdrawn children who do not participate in any activities. T9 mentioned that bullying among children appears to be increasing, and he was concerned that if psychological intervention is not provided, the victimized child may grow to become a criminal and end up in prison. This view was supported by T11, who offered that an effect of bullying is that the victim will show signs of introversion and struggle to establish social relationships with other children. T11 suggested that these children typically develop a lack of confidence, which leads to poor academic performance. This is because students who are bullied frequently miss class or fail to complete assignments. T11 further commented that victims of bullying often suffer from physical and psychological problems, including depression, stress, and anxiety. T4 commented that, “The bullied child usually shows clear signs such as isolation and depression, and this also appears in his educational achievement through a noticeable decline in his grades and appears in his immoderate behaviors and reactions that extend to the outside”.

### 3.5. Role of the School in Addressing Bullying 

Teachers in this study noted that bullying is often difficult to detect, suggesting a lack of awareness. This is because it does not usually occur when teachers are present, and the victim is usually threatened not to report the abuse. This highlights the need for communication and anti-bullying programs to be implemented. As noted earlier, most schools do not have such programs and those that do have programs often do not implement them effectively. In this regard, T14 considers it crucial that the school provides a safe environment for the child in which all types of physical or emotional violence can be addressed—whether therapeutically or preventively—so that bullying and abusive behavior can be eliminated. T14 suggested that bullies should be removed from the school. T1 shared this view, noting that teachers must monitor and record the behaviors of children they suspect may be perpetrators or victims of bullying. 

T6 reported that a student’s regular absence may indicate that he or she is a victim of bullying. This shows the need for communication between the teachers and school administration to assist teachers to address bullying. The same teacher suggested that the school should monitor the reasons for absence, and that such monitoring may help to identify cases of bullying. T6 also suggested that poor academic performance may reflect bullying victimization. Finally, T6 offered that the school should develop a structured means of identifying bodily injuries that might be the result of physical bullying. When a school discovers bullying, the participants agreed that it must be addressed quickly and decisively. T2 stated that it is important for parents to pay attention to protecting their children from bullying and that both parents and teachers should facilitate the development of self-confidence in students, along with educating them about the harms of bullying. According to T2, this education can be provided through various initiatives and types of media and should be ongoing so that important stakeholders (parents, administrators, and teachers) have the information they need to take appropriate action to address bullying.

The teachers agreed that school authorities should ensure that bullies are punished swiftly and appropriately. Also, bullying should be reported immediately, students and teachers should be alerted to the need to discuss the harms of bullying on physical, emotional, and mental health. Since victims may find it challenging to report bullying, several teachers proposed that the school should institute measures to help students report bullying, such as providing a suggestion box, along with a phone number or email to which victims or observers can report cases of bullying. Several teachers suggested that when a child is punished for bullying, other children should be made aware of this, although the school should attempt to minimize the broader or longer-term effects of punishment on the bullies. An example of such punishment could be doing community work or being deprived of free time for a specified period. T7 advocated that the school administration has a duty to host lectures and meetings with students when the school year begins and at other times during the year during which bullying is directly discussed. In such meetings, students should be told how they can report bullying, and potential bullies should be warned about the consequences of their behavior. T7 argued that the school should involve parents in these initiatives. This is because, as T9 noted, bullying behaviors call for the integrative involvement of society, home, and school. T9 offered that, when society, home, and school work together, the incidence and negative consequences of bullying can be mitigated.

## 4. Discussion

According to Olweus [2], “[a] student is being bullied or victimized when he or she is exposed, repeatedly and over time, to negative actions on the part of one or more other students” (p. 9). Olweus elaborated on “negative actions”, which refer to any attempt to intentionally injure or cause discomfort to another. Threats, taunts, teasing, calling names, hitting, pushing, kicking, pinching, or restraining an individual from physically protecting himself or herself against attack are all forms of negative actions. Olweus concluded that bullying occurs when there is an imbalance of power. Olweus explained an imbalance of power as an asymmetric power relationship. This asymmetric relationship is shown when a student exposed to adverse actions has difficulty defending himself or herself from a student or a group of students. 

Teachers in the current study emphasized that bullying is usually associated with a power imbalance, where the bully perceives or assigns himself power over another person due to factors such as the victim’s size, age, experience, socioeconomic status, strength, or help from other students. In short, bullying can be psychological or physical and involves mistreatment and harm [34].

### 4.1. The Causes of Bullying

Researchers and interested parties have addressed various potential causes of bullying. Although bullying has received some research and public policy attention and there is widespread recognition that it can cause many problems, bullying is still extraordinarily common. Researchers often attribute bullying to difficult familial environments [35]. Teachers participating in the current study mentioned that children physically or emotionally abused by parents or siblings sometimes repeat the same behaviors in the school environment. Bullies sometimes target high-achieving students because they are jealous of the attention the students receive from teachers and other students. However, these high-achieving students also can become bullies when they recognize the power of high status and scholastic recognition [35]. Some bullies target peers who have disabilities, such as speech difficulties (including stuttering). Many stutterers experience bullying, teasing, or ridicule from their peers. Victimization at school may have long-term social, emotional, and psychological effects [22,23], especially for children with special needs [24]. Furthermore, research documents the negative impacts of bullying on the academic careers of victimized students [13,14].

### 4.2. Types of Bullying

Consistent with views held by the public, teachers in the current study pointed to physical bullying as among the most common types of bullying [36,37,38,39,40,41]. Physical bullying includes the destruction of personal property, choking, biting, pinching, slapping, kicking, and hitting [38]. Verbal bullying includes the use of words to mock or threaten another child. The goal of verbal harassment is to cause embarrassment and distress in the victim. Dane et al. [37] posit that relational bullying is sometimes overlooked. This type of bullying is sometimes referred to as psychological or emotional bullying and can also take the form of social or exclusionary bullying [37]. This type of bullying can include making an individual feel unwelcome, ostracized, excluded, isolated, and ignored. It is a less overt type of bullying that can only be noticed by someone attending closely.

One form of bullying that is becoming more common is cyberbullying. Unfortunately, this type of bullying is often less visible because it happens online. Cyberbullying and in-person or face-to-face bullying methods share many features. For example, a study conducted by Raskauskas and Stoltz [39] concluded that many perpetrators of face-to-face bullying continue the bullying behavior online. The same researchers hypothesized that although the bully and victim may often be in close physical proximity, the bully may use social media platforms to ensure that more fellow students witness the bullying.

A widely adopted categorization of bullying dichotomizes these behaviors into direct and indirect bullying [40,41,42]. Direct bullying includes verbal and physical acts of oppression and intimidation, such as pushing and hitting, sarcasm and name-calling, gestures intended to threaten, and hiding or stealing the victim’s property. Indirect bullying, in contrast, often manifests as intentional social exclusion or isolation. In this regard, O’Moore and Minton [40] and Reijntjes et al. [43] included as instances of indirect bullying: criticizing, teasing, and influencing others to do the same against a particular victim. These researchers add that indirect bullying can include using the phone to make anonymous threats and to spread or write false information about the victim.

### 4.3. The Bullying Behavior of Boys and Girls 

The National Association of Social Workers [44] argued that parents, teachers, and administrators should recognize gender differences in bullying perpetration and victimization. The NASW report notes that it is more likely that girls are bullied using indirect measures when compared to boys, often constituting emotional or psychological bullying. Considering that girls are less physically aggressive than boys, their bullying is more subtle, which makes it easier for adults to overlook bullying against girls than against boys [45]. Atlas and Pepler [46] reported that most teachers perceive that physical bullying occurs more often among boys and that verbal bullying occurs more often among girls.

### 4.4. Bullying and the Role of the School 

The comments provided by teachers in the current study corroborated the results of Brank et al. [47], who suggested that bullying harms not only the person being bullied but also that the effects can be wide-ranging and have a negative impact on physical health, psychological well-being, relationships with peers and family, and schoolwork. Therefore, identifying teachers’ perceptions of bullying among primary school students may provide a wealth of knowledge about how to deal with the problem. Teachers play an important role in detecting instances of bullying and creating a school culture that makes it easier for students to break their silence in the face of bullying [48]. The school culture is defined by Coyle [49] as the symbols, signals, rules, behaviors, beliefs, and values that characterize an educational community. Previous research documents a relationship between school culture and the prevalence of bullying [50,51]. Communication about bullying should be regularly encouraged and facilitated to create awareness and begin to effectively address the problem. This is consistent with the results of the current study, which provides narrative evidence that the school is among the most important environments for influencing children. 

Teachers in this study commented on how they prevent bullying and how they might intervene when students engage in bullying. Some themes emerging from these comments include classroom announcements that highlight the importance of respecting others and the unacceptability of bullying, having separate conversations with the bully and the victim, and facilitating stress-relieving activities, such as class games. Teachers in this study suggested that the school must develop means to identify bullying and should ensure that there is a person responsible for monitoring it so that he or she can intervene when needed. This view is supported by Newman et al. [52], who posit that the teacher can play a useful role through careful observation. 

The results of the current study support the findings of Olweus [2], who suggested that in situations with appropriate adult supervision, bullying may be reduced. However, it is important to note that these studies do not indicate that adult supervision eliminates bullying. One way to ensure that the family plays its role is to educate parents and to work on bullying prevention. This includes building their children’s self-confidence and educating their children. When a child knows what bullying is, he will be able to identify it, whether it is happening to him or to someone else. This sentiment is consistent with the bullying prevention model developed by Olweus [2], which is viewed as democratic because it includes all relevant parties, including parents, students, counselors, administrators, and faculty.

Parents play an important role by providing a home environment that may help to prevent bullying, including supportive engagement coupled with effective communication between children and their parents [53,54]. Previous research suggests that parental involvement with children and with the school can help in addressing the challenges of bullying [55,56,57,58,59,60]. The teachers interviewed for the current research indicated that they sometimes had difficulty communicating to parents the importance of preventing or reducing bullying. Thus, clear guidance for facilitating teacher–parent communication, especially regarding bullying, is warranted. Understanding parents’ perspectives will help for designing strategies for addressing bullying that are sensitive to and capitalize on parental concerns and desires. This is especially important if we take into account the figures made available by UNESCO [61] indicating that around 250 million students globally are victims of bullying at school each year. 

In 2017, the Family Safety Program at the Ministry of National Guard in the Kingdom of Saudi Arabia, in cooperation with the Ministry of Education, conducted a survey on bullying, which concluded that 33% of students were victims of violence from their peers, and 15% of students were repeatedly victimized [61]. The Kingdom of Saudi Arabia launched the National Project for the Prevention of Bullying in 2016, which was presented by the Family Safety Program with the support of the Ministry of Interior and the Office of the United Nations Organization for the Arab States of the Gulf [62]. A 2019 initiative in Saudi Arabia by the Ministry of Communications and Information Technology regarding cyberbullying concluded that 18% of children and adolescents have been exposed to cyberbullying [63]. As a result, the Ministry of Education in the Kingdom of Saudi Arabia amended several laws, including student rules of conduct and attendance. Some of the actions that the Ministry of Education expects to be taken include school authorities writing to a child’s guardian about a bullying incident, making a promise to the affected student that the school authorities will not allow the incident to happen again, and apologizing to all those affected as well as repairing or replacing damaged items, where appropriate. The Ministry of Education also allows school authorities to deduct marks from bullying students. There is also an emphasis on counselling the perpetrator and victim of bullying.

In Saudi Arabia, new measures are being implemented to address bullying in schools. Research conducted and reported in 2019 by the King Abdullah Research Center concluded that bullying affects nearly 50% of children attending public schools in Saudi Arabia [63]. The 2021 report of the National Committee for Childhood in the Kingdom [64] indicates that about 57% of boys and 43% of girls are victims of bullying in Saudi Arabian public schools.

### 4.5. Limitations and Conclusions

The perceptions that teachers provided during interviews may be limited to their specific perspectives and life experiences. It is possible that participants may not want to share all or some of their opinions to protect the reputation of the schools they work for. The questions themselves may be limited because only a few studies have been conducted on teachers’ perceptions of bullying, interventions adopted to reduce it, and different types of bullying interventions. The study included a small sample, and the interviewees were all men. Certainly, future research could secure data from a larger sample of participants, including female participants. Nevertheless, insights from this study may help schools to develop effective training programs and more effectively address the many challenge of bullying. Future research could include the views of other support staff, students, and parents, who provide equally important perspectives on bullying in public primary schools in Saudi Arabia. Future research also might secure data from female teachers, to afford an investigation of teachers’ sex differences and similarities in perceptions of bullying. From the literature review and the above analysis, it is clear that bullying is still prevalent in Saudi Arabian public primary schools. It is also clear that teachers agree that bullying is a problem in schools. It is useful for teachers to be attentive to bullying behaviors as they represent a frontline for addressing these behaviors. The current study began with the goal of reporting teachers’ perceptions about bullying, noting that research links bullying to violence and other negative outcomes such as poor academic achievement and low self-esteem [62,65]. Perceptions of what teachers observe can play a crucial role in determining what actions can be taken to prevent or reduce bullying. If bullying is widespread, teachers who have documented incidents can make a compelling case regarding the seriousness of the problem. This can facilitate the development of formal strategies for addressing bullying. Teachers’ perceptions thus can include the use of behavioral assessment tools [66,67,68]. 

Based on the results of the current study, the importance of the role of teachers, schools, and parents in developing and adopting policies to prevent or reduce bullying in primary schools in the Kingdom of Saudi Arabia is evident. Eliminating school bullying requires establishing the foundations of a safe, supportive, and socially nurturing environment, and working to establish harmony, understanding, and mutual respect in the school and in classrooms. Schools will need to adopt an integrated therapeutic approach that works to involve teachers, administrators, parents, and social support networks, in addition to the bullying student himself, to address the problem, commensurate with the culture of the school and the social environment of its members.

## Data Availability

Due to the nature of this research, participants did not agree for their identified data to be shared publicly.
